# Metabolic abnormalities and survival among patients with non-metastatic breast cancer

**DOI:** 10.1186/s12885-022-10430-9

**Published:** 2022-12-29

**Authors:** Alexa S. Zimbalist, Bette J. Caan, Wendy Y. Chen, Elizabeth A. Mittendorf, Deborah A. R. Dillon, Charles Quesenberry, Elizabeth M. Cespedes Feliciano

**Affiliations:** 1 Division of Research, Kaiser Permanente Northern California, 2000 Broadway, 5Th Floor, Oakland, CA 94612 USA; 2 Channing Division of Network Medicine, Brigham and Women’s Hospital, Boston, MA 02115 USA; 3 Department of Medical Oncology, Dana Farber Cancer Institute, Boston, MA 02215 USA; 4 Division of Breast Surgery, Brigham and Women’s Hospital, Boston, MA 02215 USA; 5 Breast Oncology, Dana-Farber Brigham Cancer Center, Boston, MA 02215 USA; 6 Harvard Medical School, Boston, MA 02215 USA; 7 Department of Pathology, Brigham and Women’s Hospital, Boston, MA 02115 USA

**Keywords:** Breast cancer, Metabolic dysregulation, Dyslipidemia, Hyperglycemia, Breast cancer survival, Prognosis

## Abstract

**Background:**

Research on the impact of metabolic abnormalities on breast cancer prognosis is limited by small samples and assessment of laboratory values at a single time point, often prior to cancer diagnosis and treatment. In this population-based cohort, time-updated laboratory values were adjusted for cancer treatment to assess the association between metabolic risk factors (glucose, high-density lipoprotein cholesterol (HDL-C), low-density lipoprotein cholesterol (LDL-C), triglycerides) and breast cancer survival.

**Methods:**

13,434 women diagnosed with stage I-III breast cancer from 2005-15 at Kaiser Permanente were included. All outpatient fasting glucose, HDL-C, LDL-C, and triglyceride values from diagnosis through 2019 or death were extracted from electronic medical records. Risk of breast cancer-specific mortality was evaluated with Cox proportional hazards models adjusted for metabolic labs, demographics, body mass index, diabetes, dyslipidemia and anti-hypertensive medications, tumor characteristics (stage, ER and HER2 receptor status) and cancer treatment (use of chemotherapy, tamoxifen, and aromatase inhibitors).

**Results:**

Mean (SD) age at diagnosis was 62.3 (11.8) years. Over a median follow-up of 8.6 years, 2,876 patients died; 1,080 of breast cancer. Patients with low HDL-C (≤ 45 vs. > 45 mg/dL) had higher breast cancer-specific mortality (HR, 1.77; 95% CI, 1.53-2.05), as did those with elevated fasting glucose (> 99 vs. 60-99 mg/dL) (HR, 1.19; 95% CI, 1.03-1.37). Elevated levels of triglycerides and LDL-C were not associated with breast cancer-specific mortality.

**Conclusions:**

High fasting glucose and low HDL-C evaluated over time after cancer diagnosis were associated with higher breast cancer mortality independent of cancer treatments and changes in other metabolic risk factors. Future studies should address whether pharmacologic or lifestyle treatment of glucose and lipids after breast cancer diagnosis can optimize survival outcomes.

**Supplementary Information:**

The online version contains supplementary material available at 10.1186/s12885-022-10430-9.

## Background

Breast cancer is the second leading cause of cancer death among women in the United States [1]. Even after accounting for tumor characteristics, treatment, and other known risk factors for breast cancer mortality, there remains great heterogeneity in outcomes. Thus, identifying post-diagnosis modifiable risk factors may help improve prognosis [2–4].

Systemic metabolic dysregulation, including the highly prevalent conditions of dyslipidemia and hyperglycemia, has been implicated in breast cancer progression. Potential mechanisms include enhanced tumor cell reprogramming and chemoresistance, production of hormones and pro-inflammatory factors, and inhibited cancer cell apoptosis [4–6]. Yet, recent epidemiological studies assessing the association between hyperglycemia and breast cancer prognosis tend to focus on glucose values before or at diagnosis and often ignore the confounding influence of cancer treatment on glucose profiles and disease progression [6–8].

Dyslipidemia is also believed to influence cancer cell proliferation [2]. In particular, cholesterol’s role in cell membrane structure, cellular signaling pathways, and steroid hormone biosynthesis suggests it may be an important clinical factor in breast cancer development [9, 10]. Yet, the few studies that have assessed the relationship between lipids and prognosis have produced inconsistent results and been limited by small sample sizes and a focus on lipid profiles before lipid-altering cancer treatment [5, 11–13].

The role of metabolic dysregulation in breast cancer progression remains unclear, in part because prior studies have been conducted in small populations with assessment of laboratory values at a single time point, often at or before breast cancer diagnosis. Previous observational research often has not controlled for the influence of cancer treatments on metabolic dysregulation, even though emerging evidence suggests that chemotherapy may worsen glucose and lipid levels in breast cancer patients [14–17]. Endocrine therapies are also known to impact lipids, with prior research suggesting tamoxifen improves lipid profiles, while aromatase inhibitors worsen them [18].

This study of metabolic dysregulation and breast cancer survival is the first, to our knowledge, which assesses glucose, LDL-C, HDL-C, and triglycerides over time after breast cancer diagnosis controlling for cancer treatment.

## Methods

### Study population

This study population drew from all female Kaiser Permanente Northern California (KPNC) patients 18 years and older who were diagnosed with a first primary invasive stage I-III breast cancer from 2005 through 2014 (*n* = 21,226). Women were included if they had at least one measurement of all four labs (fasting glucose, LDL-C, HDL-C, and triglycerides) between 24 months pre-diagnosis and 6 months post-diagnosis (*n* = 13,434). Women included and excluded because of insufficient metabolic data were similar with respect to race/ethnicity, body mass index (BMI) and estrogen receptor (ER) status but differed by stage (58% vs. 49% stage I) and diabetes status (18% vs. 9%). All methods in this retrospective, data-only study were conducted in accordance with relevant guidelines and regulations approved by the Kaiser Permanente Northern California Institutional Review Board with a waiver of written informed consent.

### Metabolic measurements and start of follow-up

Outpatient fasting glucose, LDL-C, HDL-C, and triglycerides values were extracted from the electronic medical record (EMR). Baseline labs were selected as the lab value closest to diagnosis within the window of 24 months pre-diagnosis to 6 months post-diagnosis but prior to chemotherapy and radiation (baseline window). Of the four baseline laboratory values (glucose, LDL-C, HDL-C, triglycerides), the date of the most recent baseline lab measurement became the start of follow-up (follow-up start date). If all four baseline lab measurements occurred before breast cancer diagnosis, follow-up started at the diagnosis date. All metabolic measurements from baseline through follow-up were included and were dichotomized in accordance with KPNC’s clinical reference categories *(see Table **1**)*. The metabolic measurements were time-updated through follow-up and missing values were managed using last observation carried forward (LOCF) methodology. LOCF is a statistical approach to account for missing data in longitudinal studies in which a participant’s last observed value replaces their subsequent missing observations [19].

**Table 1:** Study Population Characteristics at Diagnosis of Non-Metastatic Breast Cancer (*N* = 13,434 women diagnosed at Kaiser Permanente Northern California, 2005–2015)^1^

**Age at diagnosis (years)**	62.34 (11.81)
**Race/ethnicity**	
White	8,949 (67%)
Black	981 (7.3%)
Asian/Pacific Islander	2,363 (18%)
Hispanic/Latino	999 (7.4%)
Other	131 (1.0%)
Missing	11 (< 0.1%)
**Body Mass Index Category**	
Underweight (< 18.5 kg/m^2^)	136 (1%)
Normal (18.5—< 25 kg/m^2^)	3,592 (26.7%)
Overweight (25—< 30 kg/m^2^)	4,247 (32%)
Class 1 Obesity (30—< 35 kg/m^2^)	2,689 (20%)
Class 2/3 Obesity (≥ 35 kg/m^2^)	2,193 (16%)
Missing	577 (4.3%)
**Diabetes**	2,438 (18%)
**Overall mortality**	2,876 (21%)
**Breast cancer mortality**	1,080 (8.0%)
**ER positive**	11,164 (83%)
**PR positive**	8,686 (65%)
**HER2 positive**	1,339 (10.0%)
**Stage at diagnosis**	
1	7,786 (58%)
2	4,420 (33%)
3	1,228 (9.1%)
**Surgery**	13,142 (98%)
**Radiation**	4,789 (36%)
**Chemotherapy**	5,362 (40%)
**Medications**	
Anti-hypertensives	7,681 (57%)
Statins	5,299 (39%)
Other dyslipidemia medication	514 (3.8%)
**Glucose**	
Normal (60—99 mg/dL)	7,828 (58%)
Low (< 60 mg/dL)	6 (< 0.1%)
High (> 99 mg/dL)	5,600 (42%)
**High Density Lipoprotein (LDL) Cholesterol**	
Normal (> 45 mg/dL)	10,663 (79%)
Low (≤ 45 mg/dL)	2,771 (21%)
**Low Density Lipoprotein (LDL) Cholesterol**	
Normal (< 129 mg/dL)	9,251 (69%)
High (≥ 129 mg/dL)	4,183 (31%)
**Triglycerides**	
Normal (< 199 mg/dL)	11,494 (86%)
High (≥ 199 mg/dL)	1,940 (14%)

^1^Mean (SD); n (%)

### Breast cancer-specific mortality and survival time

Death data was obtained from the KPNC death file, comprising the National Death Index and the California Department of Vital Statistics. Patients with International Classification of Diseases (ICD)-10 diagnostic code *C50: Malignant neoplasm of breast* listed as their immediate or underlying cause of death were considered to have died from breast cancer. Follow-up continued from follow-up start date to death date or December 31, 2019, whichever was earlier.

### Fixed and time-varying covariates

Age at diagnosis, race/ethnicity, tumor characteristics (stage, ER status, and human epidermal growth factor receptor 2 [HER2] status), and receipt of chemotherapy were identified from the EMR and KPNC Cancer Registry and treated as fixed covariates.

BMI, diabetes status, receipt of hormone therapies (tamoxifen and/or aromatase inhibitors), and use of dyslipidemia and anti-hypertensive medications were identified from the EMR and treated as time-varying covariates. Patients were identified as having diabetes at baseline if they had a diabetes diagnosis at any point prior to their follow-up start date. Baseline BMI was calculated using the weight measurement closest to diagnosis and the mode of the heights within the baseline window. Patients were classified as being on hormone therapies or medications at baseline if they had a prescription at any point during the baseline window. All time-varying covariates were updated from the follow-up start date through follow-up, utilizing LOCF methodology.

### Statistical analysis

Cox proportional hazards models were used to estimate hazard ratios (HRs) and corresponding 95% confidence intervals (CIs) for the association between metabolic dysregulation and breast cancer-specific mortality. Potential confounders were selected a priori and included age at diagnosis, race/ethnicity, BMI, diabetes status, tumor characteristics (stage and ER and HER2 status), cancer treatments (receipt of chemotherapy, tamoxifen and/or aromatase inhibitors), and usage of dyslipidemia and anti-hypertensive medications. A missing indicator was used for variables with missing data (*see Table **1*). The Cox proportional hazards assumption was assessed via visual examination of the Schoenfeld residuals. Stratified analyses were conducted for the following covariates: age at diagnosis (< 55 and ≥ 55 years); BMI (normal, overweight, class 1 obesity, and class 2/3 obesity as defined by the Centers for Disease Control and Prevention); diabetes status (no and yes); and ER status (negative and positive). Likelihood ratio tests were used to examine possible interactions between stratification variables and metabolic labs. A subsequent analysis of metabolic dysregulation and overall mortality was conducted to assess the potential for competing risks. All statistical analyses were performed using RStudio (version 4.0.2). Statistical significance was established with 2-sided tests with α = 0.05.

## Results

Patient characteristics are shown in Table 1. A majority (67%) were non-Hispanic white and mean (standard deviation (SD)) age at diagnosis was 62 (11.8) years. Most women were diagnosed with stage I (58%), ER-positive breast cancers (83%). A majority were overweight or obese (68%), and accordingly a considerable number had diagnosed diabetes (18%), were prescribed anti-hypertensives (57%) and/or dyslipidemia medications (43%) at baseline.

The number and temporal distribution of repeated measurements for each of the metabolic risk factors are shown in Supplemental Figures S1 and S2. The mean (SD) number of repeated measures for each metabolic risk factor (glucose, HDL-C, LDL-C, triglycerides) ranged from 5 (4) to 7 (5), and the mean (SD) time in years between first and last follow-up measurements ranged from 5 (3) to 6 (3). Categorical changes in glucose, HDL-C, LDL-C, and triglycerides from diagnosis (pre-treatment) into survivorship (the first measurement between 1.5- and 3.5-years post-diagnosis) stratified by treatment regimen are presented in supplemental Table S1. While most women remained in the same category over time, depending on the metabolic risk factor examined, up to 25% changed categories. For example, 15.2% of women moved from normal to high glucose and 7.2% from normal to low HDL-C over this period. Treatment type had little impact on these changes, except for tamoxifen where the expected decreases in LDL-C were observed.

**Fig. 1:**
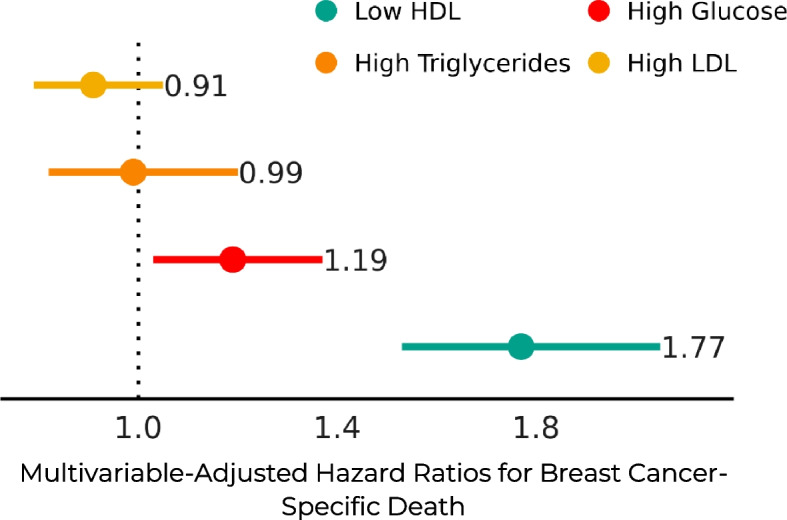
Time-Updated Metabolic Lab Values and Risk of Breast Cancer Mortality After. Stage I-III Breast Cancer Diagnosis (*N* = 13,434)^1^. ^1^High LDL (≥ 129 mg/dL); reference = normal (< 129 mg/dL). High triglycerides (≥ 199 mg/dL); reference = normal (< 199 mg/dL). High glucose (> 99 mg/dL); reference = normal (60—99 mg/dL). Low HDL (≤ 45 mg/dL); reference = normal (> 45 mg/dL)

Over a median follow-up of 8.6 years, 2,876 patients died, of whom 1,080 (37.6%) died of breast cancer. Hazard ratios and 95% CIs from Cox proportional hazards models are presented in Table 2 and supplemental Table S2. Patients with low HDL-C (≤ 45 vs. > 45 mg/dL) had higher breast cancer-specific mortality (HR, 1.77; 95% CI, 1.53–2.05) and overall mortality (HR, 1.87; 95% CI, 1.71–2.04) (*Fig. **1*). Those with high glucose (> 99 vs. 60–99 mg/dL) also had higher breast cancer-specific mortality (HR, 1.19; 95% CI, 1.03–1.37) and overall mortality (HR, 1.13; 95% CI 1.04–1.23). Elevated levels of LDL-C and triglycerides were not associated with breast cancer or overall mortality.

**Table 2:** Time-Updated Metabolic Labs and Breast Cancer Survival in Women With Stage I-III Breast Cancer (*N* = 13,434)

Characteristic	Model*		
	HR^1^	95% CI^1^	*p*-value
**Glucose**^**2**^			
Low (< 60)	5.29	2.17, 12.9	< 0.001
High (> 99)	1.19	1.03, 1.37	0.019
**HDL**^**2**^			
Low (≤ 45)	1.77	1.53, 2.05	< 0.001
**LDL**^**2**^			
High (≥ 129)	0.91	0.79, 1.05	0.2
**Triglycerides**^**2**^			
High (≥ 199)	0.99	0.82, 1.20	> 0.9

^1^HR = Hazard Ratio, *CI* Confidence Interval

^2^All labs measured in mg/dL; all reference levels are normal

*Model adjusted for age at diagnosis, race/ethnicity, stage at diagnosis, ER and HER2 status, receipt of chemotherapy, and time-updated metabolic labs, body mass index, diabetes, tamoxifen and aromatase inhibitors, and dyslipidemia medications

In subgroup analyses, the associations of the metabolic labs with survival were similar across strata defined by age (as proxy for menopausal status), BMI, diabetes, and ER status *(Table **3**)*. However, the associations of high glucose with breast cancer mortality differed by diabetes status (HR, 1.41; 95% CI, 1.21–1.65 for no diabetes; HR, 0.77; 95% CI, 0.58–1.03 for diabetes).

**Table 3:** Time-Updated Metabolic Labs and Breast Cancer Survival, Stratified by Age, Diabetes Status, Estrogen Receptor Status, and Body Mass Index

	**Low Glucose**^**1,2**^ (< 60 mg/dL)			**High Glucose**^**1,2**^ (> 99 mg/dL)			**Low HDL**^**1,2**^ (≤ 45 mg/dL)			**High LDL**^**1,2**^ (≥ 129 mg/dL)			**High Triglycerides**^**1,2**^ (≥ 199 mg/dL)		
	HR^3^	95% CI^3^	*p*-value	HR^3^	95% CI^3^	*p*-value	HR^3^	95% CI^3^	*p*-value	HR^3^	95% CI^3^	*p*-value	HR^3^	95% CI^3^	*p*-value
Pre-menopause (< 55 yrs) *(n* = *3,540; events* = *245)*	3.47	0.44, 27.1	0.24	1.24	0.89, 1.73	0.20	1.70	1.26, 2.29	< 0.001	0.85	0.64, 1.14	0.29	0.79	0.51, 1.22	0.29
Post-menopause (> = 55 yrs) *(n* = *9,894; events* = *835)*	5.97	2.19, 16.2	< 0.001	1.19	1.01, 1.39	0.035	1.78	1.50, 2.10	< 0.001	0.90	0.76, 1.06	0.19	1.03	0.84, 1.27	0.78
ER negative *(n* = *2,214; events* = *323)*	3.35	0.45, 24.6	0.24	1.14	0.87, 1.49	0.34	1.88	1.43, 2.47	< 0.001	1.15	0.90, 1.47	0.25	0.96	0.67, 1.38	0.83
ER positive *(n* = *11,164; events* = *753)*	6.64	2.44, 18.1	< 0.001	1.21	1.02, 1.43	0.027	1.74	1.47, 2.07	< 0.001	0.83	0.70, 1.00	0.044	0.97	0.78, 1.21	0.77
No Diabetes *(n* = *9,711; events* = *764)*	–-	–-	–-	1.41	1.21, 1.65	< 0.001	2.09	1.75, 2.50	< 0.001	0.91	0.78, 1.07	0.25	0.91	0.71, 1.17	0.46
Diabetes *(n* = *3,723; events* = *316)*	6.21	2.44, 15.8	< 0.001	0.77	0.58, 1.03	0.079	1.36	1.07, 1.73	0.012	0.86	0.63, 1.17	0.34	1.15	0.86, 1.53	0.34
Normal (18.5—< 25 kg/m^2^) *(n* = *3,592; events* = *268)*	2.44	0.32, 18.7	0.39	1.03	0.77, 1.38	0.83	1.82	1.30, 2.55	< 0.001	0.71	0.54, 0.95	0.022	1.29	0.84, 1.97	0.25
Overweight (25—< 30 kg/m^2^) *(n* = *4,247; events* = *309)*	16.0	4.93, 51.6	< 0.001	0.84	0.65, 1.10	0.21	1.30	0.98, 1.73	0.070	0.95	0.73, 1.23	0.69	0.69	0.47, 1.00	0.049
Class 1 Obesity (30—< 35 kg/m^2^) *(n* = *2,689; events* = *233)*	1.41	0.19, 10.3	0.73	1.07	0.79, 1.44	0.68	1.41	1.06, 1.88	0.020	1.03	0.76, 1.40	0.84	1.25	0.89, 1.75	0.21
Class 2/3 Obesity (≥ 35 kg/m^2^) *(n* = *2,193; events* = *197)*	–-	–-	–-	1.08	0.77, 1.51	0.67	1.66	1.24, 2.23	< 0.001	0.72	0.50, 1.05	0.084	0.91	0.62, 1.35	0.65

^1^All laboratory reference levels are normal

^2^Models adjusted for age at diagnosis, race/ethnicity, stage at diagnosis, ER and HER2 status, receipt of chemotherapy, and time-updated metabolic labs, body mass index, diabetes, tamoxifen and aromatase inhibitors, and dyslipidemia medications

^3^HR Hazard ratio, CI Confidence interval

## Discussion

In this study of 13,434 women with non-metastatic breast cancer from a community-based cohort, low HDL-C and elevated fasting glucose were associated with an increased risk of breast cancer-specific morality, while abnormal LDL-C and triglycerides were not. To our knowledge, this is the first study of metabolic risk factors over time after a breast cancer diagnosis that also accounts for cancer treatments and dyslipidemia medications. Prior research on metabolic dysregulation and prognosis has been inconclusive and limited by small sample sizes, short follow-up, lack of serial measurements and adjustment for cancer treatments, making few studies directly comparable.

The current study utilized time-updated measurements after breast cancer diagnosis and found that elevated glucose may be an important prognostic factor in breast cancer-specific mortality. This adverse association is similar to the results of 2 prior studies on non-diabetics that assessed pre-diagnosis and chemotherapy-related hyperglycemia with overall survival (OS) and 5-year relapse-free survival (RFS) [7, 8]. 2 prior studies that found null associations between at-diagnosis hyperglycemia and various breast cancer outcomes did not adjust for diabetes status nor cancer treatment which may increase glucose levels and impact prognosis [7, 20, 21]. In contrast, this study’s stratified analyses adjusted for cancer treatment and found an adverse association of elevated glucose and breast cancer-specific mortality only among non-diabetics. The apparent inverse association observed among women with diabetes is potentially due to antidiabetic medications used to lower high glucose levels.

In addition to elevated glucose, low HDL-C evaluated over time was associated with breast cancer-specific mortality. This finding is consistent with 2 prior smaller studies that evaluated preoperative and at-diagnosis low HDL-C with overall survival, 1 of which only found a significant association among those with triple-negative breast cancers.[5, 20] In contrast to the current study’s findings, null associations with overall and breast-cancer specific mortality, as well as disease-free survival (DFS) and breast cancer recurrence have been reported [13, 22–24]. A potential explanation is that those studies were smaller and assessed HDL-C at a single time point. Moreover, only 1 of those studies adjusted for chemotherapy, whereas this study evaluated HDL-C over a long follow-up period and accounted for the effects of chemotherapy, tamoxifen, aromatase inhibitors, and dyslipidemia medications, all of which affect lipid profiles [18, 25].

Unlike elevated glucose and low HDL-C, high triglycerides and LDL-C were not associated with an increased risk of breast cancer-specific mortality. The null effect of triglycerides is in accordance with the majority of published studies on triglycerides and breast cancer outcomes, except for 1 that found high triglyceride levels to be protective and 1 other that suggested the opposite [4, 5, 13, 20–24]. Of the very few studies that have assessed LDL-C and prognosis, 2 have reported null associations with OS and DFS, while 1 found at-diagnosis high LDL-C was associated with worse DFS at 25 months follow-up [12, 23, 24]. The adverse association was reported in a study with a small sample size and short follow-up period that excluded women on dyslipidemia and/or anti-diabetic medications, yet, did not account for the lipid-altering effects of chemotherapy and endocrine therapy as the current study did [12, 18, 25]. Additionally, epidemiological evidence suggests that statin use among breast cancer patients can target cholesterol metabolism and decrease mortality risk, which could provide a potential explanation for why this study, which adjusted for use of statins over time, did not detect associations with LDL-C [26].

The exact mechanisms by which hyperglycemia and lipids affect breast cancer progression are still being studied. The adverse association found in this study between hyperglycemia and breast cancer-specific mortality is substantiated by current literature that hyperglycemia may promote tumor cell proliferation, invasion, and migration through various pathways, including the “Warburg” effect resulting in increased glucose consumption by cancer cells, activation of epidermal growth factor and insulin receptors, and production of pro-inflammatory factors. Hyperglycemia is also thought to alter the tumor microenvironment, inhibit cancer cell apoptosis, and increase the chemoresistance of tumor cells [6, 27].

Excess lipid biosynthesis may also influence tumor cell proliferation through increased tumor cell metabolic reprogramming, including increased: building materials for cell membrane components, energy via oxidation of fatty acids, and signaling molecules that influence oncogenic pathways [28–31]. Although hypotheses as to how HDL-C specifically impacts tumor progression are limited, this study’s findings that low HDL-C worsens breast cancer-specific mortality are in accordance with theories that low HDL-C increases activity of pro-inflammatory cytokines and decreases regulation of angiogenesis which could encourage tumor metastasis [4].

Strengths of the study include being the first large study evaluating multiple metabolic exposures and breast cancer-specific survival with a long follow-up time, and incorporation of time-varying exposures post cancer diagnosis. Furthermore, robust multivariate models were used to control for multiple potential confounders, including cancer characteristics and treatment and dyslipidemia medications. Limitations include exclusion of women with insufficient metabolic data; while women in the study were more likely to be diabetic, other notable differences in their characteristics were not detected and stratified analyses by diabetes status were conducted. In addition, clinically collected EMR data was used to characterize metabolic risk factors; thus, the timing of data collection was not perfectly aligned with diagnosis or at regular follow-up intervals. However, the analytic approach enabled us to update metabolic risk factors over time, a novel strength.

## Conclusions

The results of this study indicate that elevated glucose and low HDL-C evaluated over time after breast cancer diagnosis is associated with worse breast cancer-specific mortality, after controlling for cancer treatments, dyslipidemia medications, and changes in other metabolic risk factors. Although this study did not detect differences in associations by ER status, future studies by breast cancer subtype are necessary to understand the impact of metabolic dysregulation on the metabolic reprogramming of different tumor types [3, 32]. Given the preclinical literature indicating metabolism may influence tumor progression and the current study’s findings of the significant role of elevated glucose and low HDL-C on breast cancer specific-mortality, future studies should address whether pharmacologic or lifestyle treatment of glucose and lipids after breast cancer diagnosis can optimize survival outcomes.

## Supplementary Information

**Additional file 1:** **Table S1.** Change in laboratory values from diagnosis to post-treatment (1.5-3.5 years post-diagnosis).

**Additional file 2: Table S2.** Time-Updated Metabolic Labs and Overall Survival in Women With Stage I-III Breast Cancer (N = 13,434; Events = 2,876)**.**

**Additional file 3:** **Figure S1.** Number of Repeated Measurements by Metabolic Risk Factor.

**Additional file 4:** **Figure S2.** Distribution of time between first and last follow-up measurements among women with at least 2 follow-up measurements.

## Data Availability

The datasets generated and/or analyzed during the current study are not publicly available due to being generated based on information collected during clinical care but are available in de-identified form from the corresponding author on reasonable request at the study’s close.

## Abbreviations

HDL-C: High-density lipoprotein cholesterol
LDL-C: Low-density lipoprotein cholesterol
KPNC: Kaiser permanente Northern California
BMI: Body mass index
ER: Estrogen receptor
EMR: Electronic medical record
ICD: International classification of diseases
HER2: Human epidermal growth factor receptor 2
LOCF: Last observation carried forward
HR: Hazard ratio
CI: Confidence interval
SD: Standard deviation
OS: Overall survival
RFS: Relapse-free survival
DFS: Disease-free survival
