# Supplementary material for: Metabolic abnormalities and survival among patients with non-metastatic breast cancer
Source: BMC Cancer. 2022 Dec 29;22:1361. doi: 10.1186/s12885-022-10430-9 (PMC9801571; doi:10.1186/s12885-022-10430-9)
Supplement: Supplementary file 1 — Additional file 1: Table S1. Change in laboratory values from diagnosis to post-treatment (1.5-3.5 years post-diagnosis). [file 12885_2022_10430_MOESM1_ESM.docx]

| Table S1. Change in laboratory values from diagnosis to post-treatment (1.5-3.5 years post-diagnosis) | | | | | | | |
| --- | --- | --- | --- | --- | --- | --- | --- |
|  | **Overall** | **Chemotherapy** | **Aromatase inhibitor** | **Tamoxifen** | **Chemotherapy + aromatase inhibitor** | **Chemotherapy + tamoxifen** | **No chemotherapy or hormonal therapy** |
| **Glucose (n=8163)^1^** | | | | | | | |
| Normal-->Normal | 3371 (41.3%) | 417 (46.2%) | 1183 (36.7%) | 393 (49.6%) | 666 (39.2%) | 290 (52.4%) | 422 (42.4%) |
| Normal-->High | 1238 (15.2%) | 136 (15.1%) | 503 (15.6%) | 111 (14%) | 273 (16.1%) | 72 (13%) | 143 (14.4%) |
| High-->High | 2766 (33.9%) | 257 (28.5%) | 1240 (38.5%) | 201 (25.3%) | 588 (34.6%) | 143 (25.9%) | 337 (33.8%) |
| High-->Normal | 788 (9.7%) | 92 (10.2%) | 296 (9.2%) | 88 (11.1%) | 170 (10%) | 48 (8.7%) | 94 (9.4%) |
| **HDL-C (n=9580)^1^** | | | | | | | |
| Normal-->Normal | 6857 (71.6%) | 755 (72.2%) | 2712 (72.1%) | 669 (73.4%) | 1396 (69.2%) | 434 (67.7%) | 891 (74.1%) |
| Normal-->Low | 691 (7.2%) | 54 (5.2%) | 282 (7.5%) | 75 (8.2%) | 154 (7.6%) | 56 (8.7%) | 70 (5.8%) |
| Low-->Low | 1323 (13.8%) | 143 (13.7%) | 519 (13.8%) | 114 (12.5%) | 293 (14.5%) | 90 (14%) | 164 (13.6%) |
| Low-->Normal | 709 (7.4%) | 94 (9%) | 250 (6.6%) | 54 (5.9%) | 173 (8.6%) | 61 (9.5%) | 77 (6.4%) |
| **LDL-C (n=9030)^1^** | | | | | | | |
| Normal-->Normal | 5275 (58.4%) | 506 (51.2%) | 2116 (59.3%) | 547 (64.4%) | 1058 (55.2%) | 375 (62.7%) | 673 (60.7%) |
| Normal-->High | 904 (10%) | 142 (14.4%) | 354 (9.9%) | 33 (3.9%) | 228 (11.9%) | 35 (5.9%) | 112 (10.1%) |
| High-->High | 1547 (17.1%) | 210 (21.3%) | 613 (17.2%) | 86 (10.1%) | 379 (19.8%) | 65 (10.9%) | 194 (17.5%) |
| High-->Normal | 1304 (14.4%) | 130 (13.2%) | 487 (13.6%) | 183 (21.6%) | 251 (13.1%) | 123 (20.6%) | 130 (11.7%) |
| **Triglycerides (n=9123)^1^** | | | | | | | |
| Normal-->Normal | 6963 (76.3%) | 772 (77.5%) | 2704 (75.2%) | 667 (77.5%) | 1472 (76%) | 461 (75.3%) | 887 (79.3%) |
| Normal-->High | 713 (7.8%) | 56 (5.6%) | 267 (7.4%) | 90 (10.5%) | 148 (7.6%) | 73 (11.9%) | 79 (7.1%) |
| High-->High | 781 (8.6%) | 76 (7.6%) | 347 (9.6%) | 64 (7.4%) | 168 (8.7%) | 47 (7.7%) | 79 (7.1%) |
| High-->Normal | 666 (7.3%) | 92 (9.2%) | 280 (7.8%) | 40 (4.6%) | 149 (7.7%) | 31 (5.1%) | 74 (6.6%) |
| ^1^Glucose – Normal: 60-99; High: >99 (mg/dL) | | | | | | | |
| HDL-C – Normal: >45; Low: ≤45 (mg/dL) | | | | | | | |
| LDL-C – Normal: <129; High: ≥129 (mg/dL) | | | | | | | |
| Triglycerides – Normal: <199; High: ≥199 (mg/dL) | | | | | | | |
| ^2^1,862 women did not receive chemotherapy or hormonal therapy, but 96% of them had surgery and 36% received radiation. | | | | | | | |
