# Supplementary material for: Metabolic abnormalities and survival among patients with non-metastatic breast cancer
Source: BMC Cancer. 2022 Dec 29;22:1361. doi: 10.1186/s12885-022-10430-9 (PMC9801571; doi:10.1186/s12885-022-10430-9)
Supplement: Supplementary file 2 — Additional file 2: Table S2. Time-Updated Metabolic Labs and Overall Survival in Women With Stage I-III Breast Cancer (N = 13,434; Events = 2,876). [file 12885_2022_10430_MOESM2_ESM.docx]

Table S2. Time-Updated Metabolic Labs and Overall Survival in Women With Stage I-III Breast Cancer (N = 13,434; Events = 2,876)

| **Characteristic** | **Model*** | | |
| --- | --- | --- | --- |
|  | HR^1^ | 95% CI^1^ | p-value |
| **Glucose^2^** |  |  |  |
| Low (<60) | 4.71 | 2.59, 8.58 | <0.001 |
| High (>99) | 1.13 | 1.04, 1.23 | 0.005 |
| **HDL^2^** |  |  |  |
| Low (≤45) | 1.87 | 1.71, 2.04 | <0.001 |
| **LDL^2^** |  |  |  |
| High (≥129) | 0.81 | 0.74, 0.89 | <0.001 |
| **Triglycerides^2^** |  |  |  |
| High (≥199) | 0.96 | 0.86, 1.08 | 0.54 |
| ^1^HR = Hazard Ratio, CI = Confidence Interval  ^2^All labs measured in mg/dL; all reference levels are normal  *Model adjusted for age at diagnosis, race/ethnicity, stage at diagnosis, ER and HER2 status, receipt of chemotherapy, and time-updated metabolic labs, body mass index, diabetes, tamoxifen and aromatase inhibitors, and dyslipidemia medications | | | |
