# Supplementary figures and images for: Metabolic abnormalities and survival among patients with non-metastatic breast cancer
Source: BMC Cancer. 2022 Dec 29;22:1361. doi: 10.1186/s12885-022-10430-9 (PMC9801571; doi:10.1186/s12885-022-10430-9)

Figure S1. Number of Repeated Measurements by Metabolic Risk Factor

**
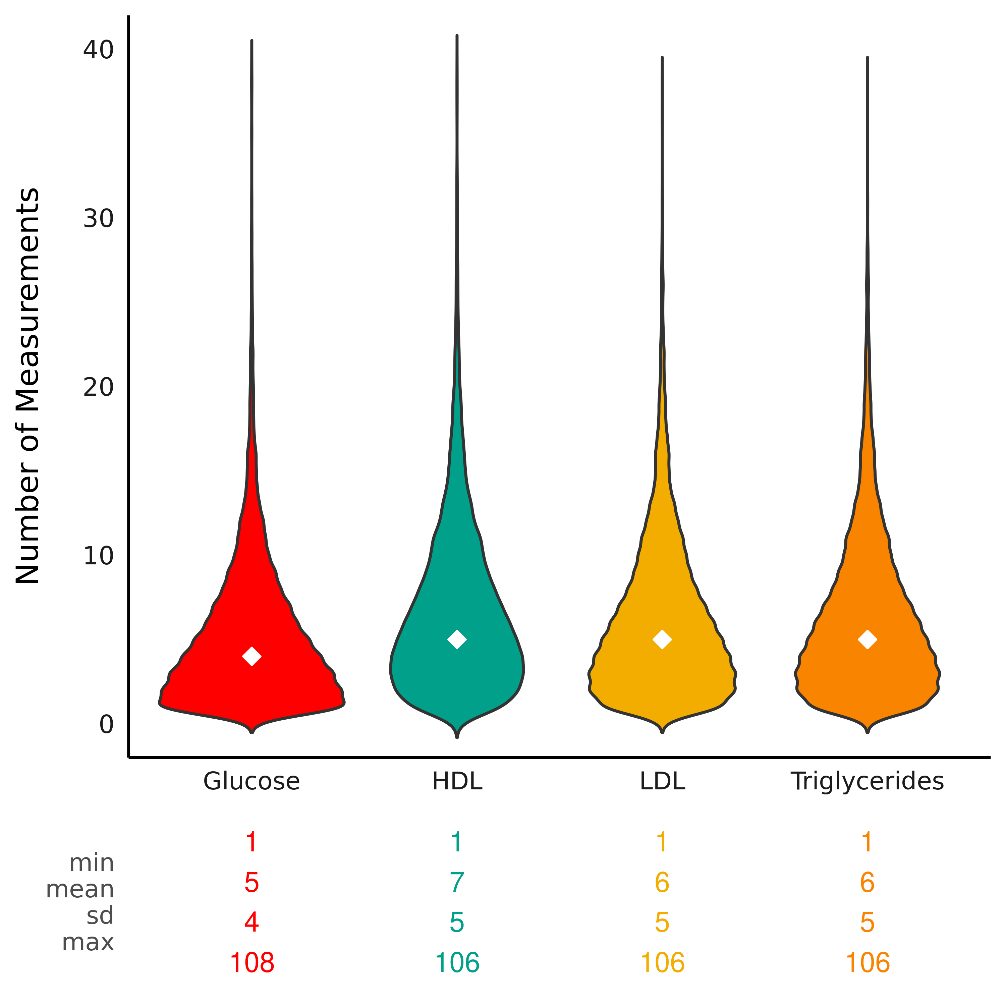
**

Supplement: Supplementary file 3 — Additional file 3: Figure S1. Number of Repeated Measurements by Metabolic Risk Factor. [file 12885_2022_10430_MOESM3_ESM.docx]
