# Supplementary material for: Metabolic abnormalities and survival among patients with non-metastatic breast cancer
Source: BMC Cancer. 2022 Dec 29;22:1361. doi: 10.1186/s12885-022-10430-9 (PMC9801571; doi:10.1186/s12885-022-10430-9)

Figure S2. Distribution of time between first and last follow-up measurements among women with at least 2 follow-up measurements


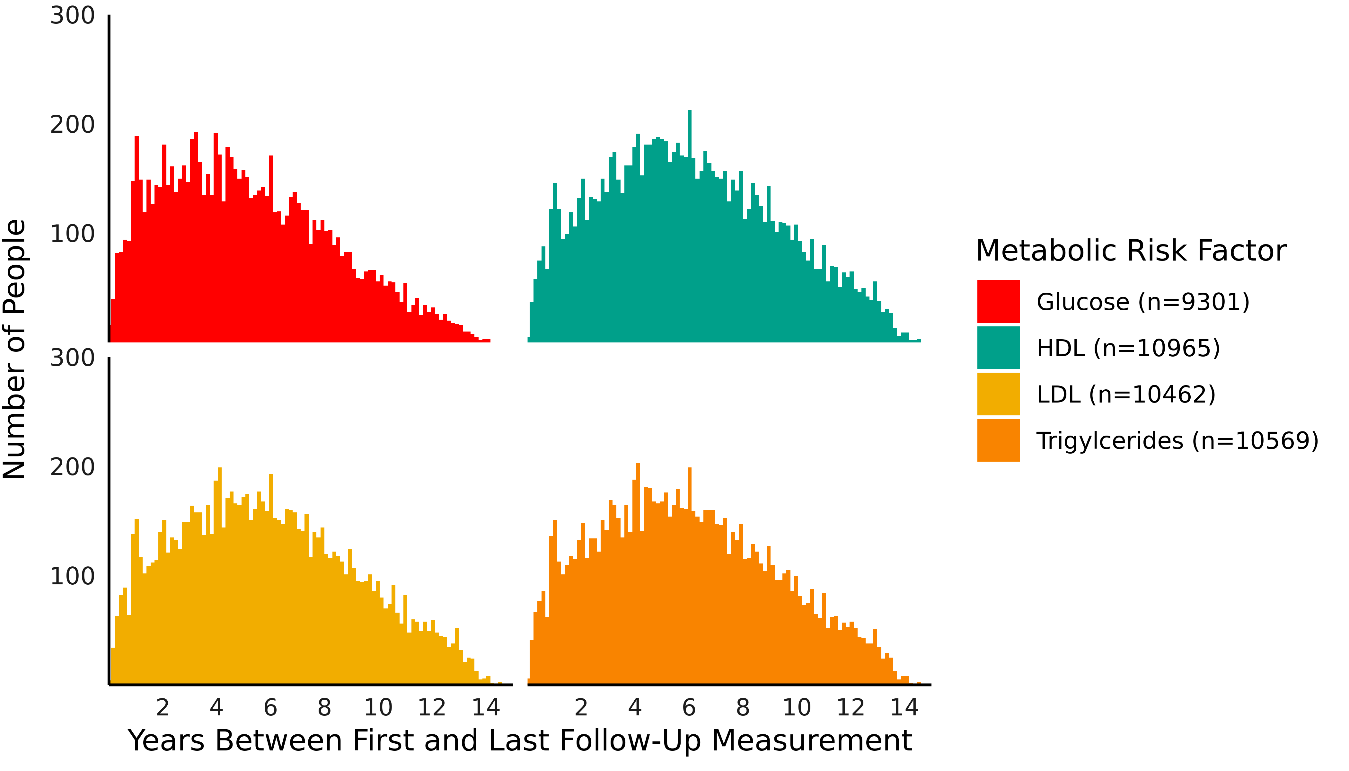

Supplement: Supplementary file 4 — Additional file 4: Figure S2. Distribution of time between first and last follow-up measurements among women with at least 2 follow-up measurements. [file 12885_2022_10430_MOESM4_ESM.docx]
